# Relationship between periodontal disease and preterm birth. A systematic review and meta-analysis

**DOI:** 10.4317/medoral.26830

**Published:** 2024-10-13

**Authors:** José María Montoya-Carralero, Rebeca Ávila-Villasmil, Arturo Sánchez-Pérez, Alfonso Jornet-García, Enrique Terrer-Alonso, María José Moya-Villaescusa

**Affiliations:** 1 Department of Periodontology. Faculty of Medicine and Dentistry. University of Murcia. Spain; 2 Dental Graduate. University of Murcia; 3 Dental Graduate. Máster´s student of Periodontology and Implantology. University of Murcia

## Abstract

**Background:**

Periodontal disease (PD) is a chronic inflammatory disease of multifactorial origin that affects the supporting tissues of the tooth. According to WHO in 2022, severe periodontal disease will affect around 19% of the adult population worldwide. Its risk factors include the presence of periodontopathogenic bacteria in biofilm and the susceptibility of the host’s immune system, among others. Preterm birth is defined as birth occurring before 37 weeks of gestational age. It also has a multifactorial origin and it’s associated with risk factors such as intrauterine and extrauterine infections. There is a possibility that periodontal disease in pregnant women increases the risk of preterm birth through hematogenous pathways or the presence and intervention of inflammatory mediators.

**Material and Methods:**

Through a systematic review of existing scientific articles from 2014 to 2024, five randomized clinical trials were selected, including a total of 1984 pregnant patients diagnosed with periodontal disease. Half of these patients received non-surgical treatment, while the other half did not, aiming to evaluate a possible association between periodontal disease and/or its treatment and the occurrence of preterm birth. The risk of bias was assessed using the Cochrane “RoB 2” tool, and finally, a meta-analysis was conducted to compare the results obtained in the selected studies.

**Results:**

Four articles showed a trend favoring non-surgical periodontal treatment as a preventive measure for preterm birth. These articles accounted for 92.2% of the total weight, while the remaining 7.85% corresponded to the single article that did not favor the treatment. However, none of the articles showed statistically significant results.

**Conclusions:**

There is no demonstrated association between periodontal disease in pregnant women and the incidence of preterm birth. On the other hand, there is a slightly favorable trend towards non-surgical periodontal treatment during pregnancy as a measure to reduce the incidence of preterm birth, but it’s not statistically significant. To obtain a definitive answer, more randomized clinical trials following similar study and design parameters are needed.

** Key words:**Periodontal disease, preterm birth, periodontal treatment, adverse pregnancy outcomes.

## Introduction

Periodontal disease (PD) is a chronic inflammatory disease of multifactorial origin that affects the supporting tissues of the tooth. According to WHO in 2022, severe periodontal disease will affect around 19% of the adult population worldwide. Its risk factors include the presence of periodontopathogenic bacteria in biofilm and the susceptibility of the host’s immune system, among others. Its treatment can be surgical or non-surgical, such as chemical and/or mechanical control of the biofilm through subgingival instrumentation. Preterm birth is defined as birth occurring before 37 weeks of gestational age. It also has a multifactorial origin and it’s associated with risk factors such as intrauterine and extrauterine infections. There is a possibility that periodontal disease in pregnant women increases the risk of preterm birth through hematogenous pathways or the presence and intervention of inflammatory mediators.

The term periodontal medicine was described by Offenbacher in 1996 as the "discipline that focuses on exploring the associations between periodontal diseases and systemic diseases, as well as their biological plausibility in human populations and animal models" (1). In 2013, the European Federation of Periodontology (EFP) and the American Academy of Periodontology (AAP) first convened in a Workshop to review the large number of publications linking periodontal disease with certain systemic conditions. The conditions that, to some extent, presented stronger evidence or biological plausibility were diabetes mellitus, certain cardiovascular diseases, and specific adverse pregnancy outcomes (APOs) (2).

Although this Workshop took place in 2013, studies on the relationship between periodontitis and systemic diseases began to be published as early as the late 19th century (3).

In this context, it is important to recognize the hormonal changes that women undergo during pregnancy, especially the temporary increase in estrogen and progesterone that occurs in the third trimester, preparing the body for childbirth. This occurs through the increase in inflammatory mediators, which not only have a positive function during this stage but also enhance the immune response, facilitating the establishment of inflammatory processes throughout the body (4), including those that occur during periodontitis.

The presence of inflammatory mediators such as TNF-α and IL-6, associated with intrauterine infections or an exacerbated intrauterine inflammatory response, is directly related to the induction of contractions or membrane rupture, which, if occurring before 37 weeks of gestation or depending on the maturity level of the fetus, is considered preterm labor or even spontaneous abortion.

Two pathogenic mechanisms or pathways are described through which the appearance of different conditions may develop APOs (5):

1. Direct pathway: when different microorganisms invade the feto-placental unit, either via the bloodstream from the oral cavity or via an ascending route from the genitourinary tract.

2. Indirect pathway: when inflammatory mediators produced locally (for example, in periodontal tissues during the establishment of periodontal disease), such as C-reactive protein, increase the systemic inflammatory response by circulating to the liver, affecting the feto-placental unit.

The direct route is considered the most plausible pathway through which the transmission of microorganisms from periodontal tissues to the uterus may occur. Various studies, such as the one by Figuero, *et al*. in 2020, associate the presence of antibodies against oral microorganisms found in umbilical cord blood and maternal serum with the exposure of the fetoplacental unit to periodontal infections (5).

However, there is very little evidence to confirm this theory, although periodontal pathogens such as *P. *gingivalis**, T. denticola, T. Forsythia y *F. nucleatum* have been associated with various adverse pregnancy outcomes (APOs), such as fetal death and spontaneous abortion, some of these microorganisms have also been found in the fetoplacental unit of women who did not experience preterm birth (PTB) or any other APO.

The same occurs with the inflammatory mediators related to PD (IL-1β, IL-6, PGE2, TNF-α, reactive protein, 8-isoprostanes, soluble intracellular adhesion molecule-1, matrix metalloproteinases, fibronectin, and ⲁ-fetoprotein), which have also been found in maternal serum, umbilical cord blood, and amniotic fluid of women who have or have not experienced various APOs.

There is a great heterogeneity in the findings over recent years regarding the relationship between periodontal disease and its treatment with the incidence of preterm births, due to the great variety in the definitions of periodontal disease, type and extent of periodontal examination, inclusion criteria, sample selection, and other factors.

Given the possibility that the presence of PD in pregnant women may increase the risk of PTB and due to the high morbidity and mortality rates that this complication represents for both mothers and newborns, it is necessary to evaluate in more detail the role that the presence of PD plays or how its treatment might influence the reduction in the incidence of PTB.

## Material and Methods

The search included randomized clinical trials (RCTs) published between 2014 and 2024 in various databases in English and Spanish, relating periodontal disease in pregnant women to preterm birth and/or its treatment. Animal studies, books, documents, and reviews published more than 10 years ago in languages other than English and Spanish were excluded.

Eligibility criteria were established following the PICO model (patient, intervention, comparison, and outcome) for clinical research methodology.

P: pregnant patients with periodontal disease who had preterm births.

I: preterm and/or premature birth associated with the presence of periodontal disease in pregnant patients.

C: with pregnant patients with periodontal disease who did not have preterm and/or premature births.

O: pregnant patients with periodontal disease are more likely to experience preterm or premature births.

Resulting question: Does the presence of periodontal disease and/or its treatment in pregnant women affect the likelihood of preterm births?.

The search strategy employed three databases: PubMed, Cochrane Library, and Scielo. The keywords used were “periodontal disease,” “periodontitis,” “periodontology,” “periodontal treatment,” “preterm birth,” “premature birth,” and “obstetrics,” combined with the Boolean operators “OR” and “AND.” The combination used in this search strategy for each database was: ((Periodontal disease) OR (Periodontitis) OR (Periodontology) OR (Periodontal treatment)) AND ((Preterm birth) OR (Premature birth) OR (Obstetrics)).

The risk of bias in the selected randomized clinical trials was assessed using the Cochrane tool “RoB 2,” resulting in one of three outcomes: low, some concerns, or high. “Low” corresponds to low risk of bias, “some concerns” indicates risk of bias in at least one domain, and “high” denotes risk of bias in at least one domain.

The Review Manager v.5.4.1 software was used for interpreting the statistical results.

## Results

The search was conducted on January 19, 2024, by two independent reviewers (R.A.-V. and E.T.-A.), resulting in a total of 155 articles. There was no disagreement in the selection of articles as the inclusion and exclusion criteria were established in advance. On May 6, 2024, a new search was performed under the same parameters in the same databases, aiming to identify any new articles published since the initial search date that might be relevant for this meta-analysis. However, this search did not yield any relevant articles, and the initial selection was maintained.

**Figure 1:**
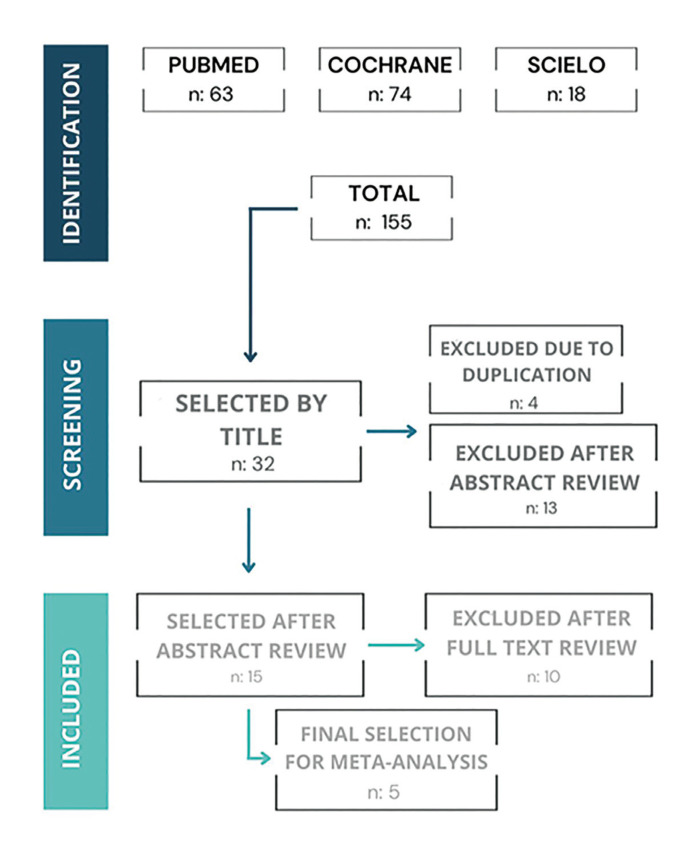
Flow chart.

Out of the 155 articles (Fig. 1), 32 were selected based on their titles and 4 were excluded due to duplication, resulting in an initial selection of 28. After reviewing each abstract, 13 articles were excluded for not meeting the eligibility criteria, leaving a total of 15 articles for full-text review. After this review, 10 articles were excluded, resulting in a final selection of 5 articles for the meta-analysis using the statistical program Review Manager v.5.4.1. The description and results obtained in the selected studies are shown in Table 1 and Table 2.

**Figure 2:**
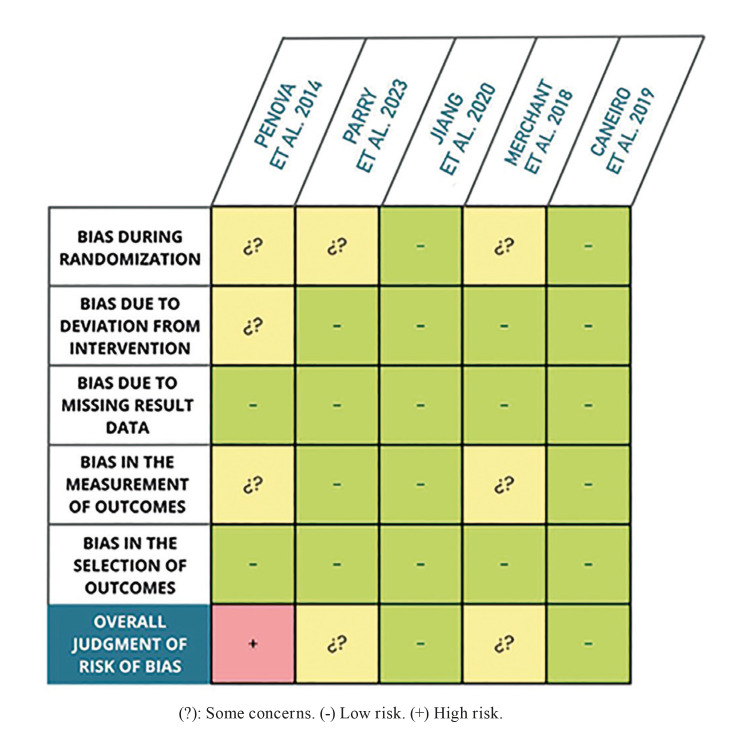
Results of the risk of bias assessment with RoB 2 - Cochrane.

The assessment of the risk of bias in the selected randomized clinical trials was conducted using the tool provided by Cochrane, "RoB 2" (Fig. 2).

Subsequently, Review Manager v.5.4.1 was used for the interpretation of the results.

**Figure 3:**
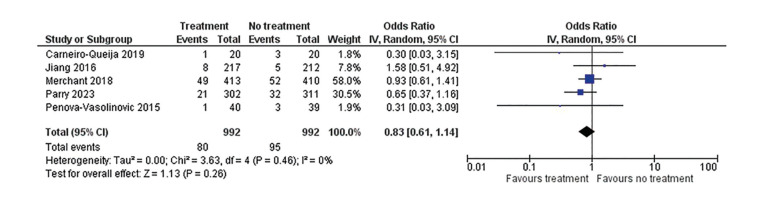
Forest Plot. Variability among studies and overall effect estimation using Review Manager v.5.4.1.

In this systematic review with meta-analysis on the relationship between maternal periodontal disease and its treatment with the occurrence of preterm birth, a total of 1,984 pregnant patients were included, all diagnosed with some form of periodontal disease. Of these 1,984 patients, 992 were in the control groups and the remaining 992 were in the experimental groups (Fig. 3). In the experimental groups, 80 patients had preterm birth (8.06%), while in the control groups, 95 patients had preterm birth (9.5%).

The studies by Caneiro *et al*. in 2019 (6) and Penova-Vaselinovic *et al*. in 2015 (7), despite having the smallest sample sizes among the five studies (*n*=20 and *n*=40 respectively, out of a total of 992 participants in the treatment group), demonstrate the highest results favoring periodontal disease treatment as a preventive measure against PTB. These two studies also exhibit some heterogeneity, as indicated by the wide confidence intervals (horizontal lines).

The study by Merchant *et al*. in 2018 (8), with the largest sample size in the treatment group of all the studies (*n*=413), is the study whose results are closest to the point of no effect, despite showing that the experimental group had a higher fetal survival rate than the control group. This study showed the least heterogeneity among the five selected.

The study by Parry *et al*. in 2023 (9), has the second-largest sample size among all treatment groups (*n*=302 patients). Its findings are close to the point of no effect but to a lesser extent than the study by Merchant *et al*. in 2018 (8). It also demonstrated low heterogeneity.

Finally, the study by Jiang *et al*. in 2016 (10), with a sample size of *n*=217 patients in the experimental group, shows results favoring no treatment, interpreted as a higher risk of PTB in the experimental group. In terms of heterogeneity, it ranks third.

The diamond represents the estimated average effect of all studies included in this meta-analysis. In this case, there is a slight general trend favoring treatment with an OR of 0.83, 95% CI [0.61-1.14]. However, since it slightly crosses the line of no effect, the combined effect can be interpreted as not statistically significant.

Although there is slight variability among the confidence intervals of the five selected studies when compared to each other, it is considered that there is no heterogeneity, as I² is 0%, Cochran's Q is high, greater than 0.05 (*P*=0.26), and Tau² is 0.00, indicating statistical homogeneity.

**Figure 4:**
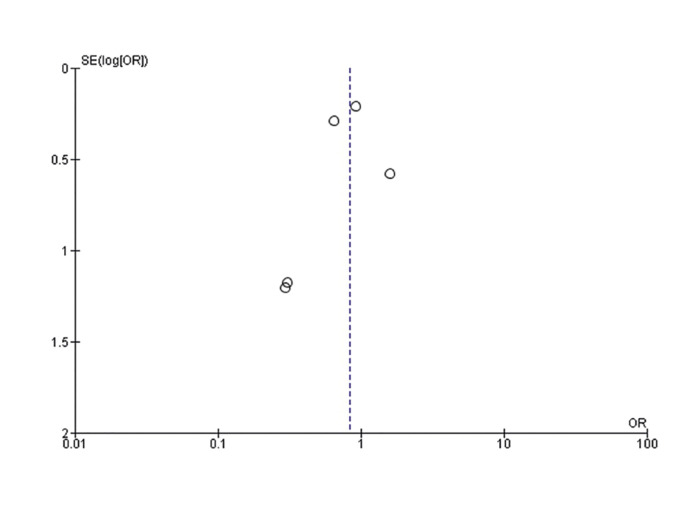
Funnel Plot. Risk of bias assessment of the studies using Review Manager v.5.4.1.

The results obtained in the risk of bias assessment using the RoB - Cochrane tool is represented by the Funnel Plot of Fig. 4.

Three of the five studies are concentrated at the top of the graph, indicating a lower risk of bias, while the remaining two studies are located at the bottom of the graph, indicating a higher risk of bias.

The asymmetric shape of the funnel plot indicates a high risk of publication bias, and the skew towards one side and the presence of empty spaces suggest a lack of published studies.

## Discussion

Although the aim of this study was to assess the potential relationship between periodontal disease and/or its treatment in pregnant patients and the incidence of preterm births through a meta-analysis, it did not manage to demonstrate a clear association because statistically significant results were not obtained individually in any of the reviewed articles.

It is crucial to recognize and discuss the inherent limitations of this study as they provide a realistic framework for interpreting the findings and highlight areas that might require attention in future research where a concrete relationship between PD and PTB might be demonstrated.

During the search and selection process of the articles, there was a disparity in the inclusion criteria applied across different studies, with significant variations in sample size, socioeconomic characteristics, ethnicity, presence of systemic diseases, and habits such as smoking and/or alcohol use. Additionally, gestational age varied widely in these studies, from the beginning of pregnancy to the end of the second trimester.

A very important factor to consider is that despite the existence of the 2017 Classification of Periodontal and Peri-implant Diseases (11), mentioned at the beginning of this study, only the article by Caneiro *et al*. in 2019 (6) followed its parameters for establishing a standard diagnosis of periodontal disease, while the other selected articles did not. This may be because most were designed or conducted before the classification was published or simply did not take it into account. These variations in diagnosing periodontal disease can complicate the interpretation of results and even pose a risk of bias, as concluded in the 2013 AAP-EFP Workshop on the relationship between periodontal diseases (2) and certain systemic, which led to the publication of the aforementioned classification a few years later.

On the other hand, despite disparities among different classifications of preterm birth, with the WHO defining it as extremely preterm (less than 28 weeks), very preterm (28 to 32 weeks), and moderately to late preterm (32 to 37 weeks) and the American College of Obstetricians and Gynecologists defining it simply as birth before 37 weeks of gestation, all the selected articles for this meta-analysis defined the criterion for considering a preterm birth as one occurring before 37 weeks of gestation.

Another limitation to consider is the variability in the quality of the selected articles. Applying the Cochrane "RoB 2" tool, the methodological quality of most studies included in this meta-analysis is relatively questionable. The study by Penova-Vaselinovic *et al*. in 2015 (7), Merchant *et al*. in 2018 (8) and Parry *et al*. in 2023 (9), scored lower in the first domain due to differences in randomization methods. Such deviations lead to a reduction in scores, resulting in an overall high risk of bias (as in the Penova-Vaselinovic *et al*. study in 2015 (7))or "some concerns" (as in the Parry *et al*. 2023 and Merchant *et al*. 2018 studies (8)), which does not directly invalidate the results but indicates the need for cautious interpretation.

Conversely, this tool also allowed for the evaluation of high methodological quality articles, such as the study by Jiang *et al*. in 2016 (10). and the study by Caneiro *et al*. in 2019 (6),which received a low-risk judgment, providing greater confidence in their results. Their methodology can serve as a model for future research to obtain more reliable results and represent a true advance in this context.

Although these limitations are acknowledged, their impact on the interpretation of the results of this meta-analysis was not significant, as the heterogeneity of results was null (I² = 0%) and all included studies essentially reached the same conclusion: the results were not statistically significant to confirm or refute the relationship between periodontal disease in pregnant women and/or its treatment and the incidence of PTB.

In studies where scaling and root planing (SRP) were performed during the second trimester of pregnancy (6,7,8) a lower incidence of preterm birth was found in the experimental group. As demonstrated by Penova-Vaselinovic *et al*. in 2015 (7), lower levels of inflammatory mediators such as IL-1β, IL-10, IL-12p70, and IL-6 were found in the crevicular fluid of pregnant patients with periodontal disease who received SRP (experimental group) compared to those who did not (control group). However, the latter group had lower levels of MCP-1 and TNF-α. As Offenbacher indicated in 1996, these inflammatory mediators (IL-1β, PGE2, TNF-α, and IL-6) are directly related to the pathogenesis of periodontal disease (1), and although IL-1β, PGE2, and TNF-α levels naturally increase as pregnancy approaches the third trimester, exaggerated increases have been associated with various adverse pregnancy outcomes, especially preterm birth (12).

In the study by Merchant *et al*. in 2018 (8), where SRP was performed during the second trimester of pregnancy along with oral hygiene instructions, a higher occurrence of intrauterine death and bleeding due to spontaneous abortion was found. Including these values in statistical analysis and applying average causal effect on survival (SACE) determined that the experimental group had a lower incidence of preterm birth, relating to the non-surgical periodontal treatment increasing fetal survival rates (8). This corresponds with the conclusions obtained in the Michalowicz *et al*. 2006 study, which observed a slight reduction in spontaneous abortion and fetal death in women who received periodontal treatment (13).

In the study by Caneiro *et al*. in 2019 (6), despite lower preterm birth rates in the experimental group, no statistically significant differences were found to confirm or refute the relationship between non-surgical periodontal treatment during pregnancy and preterm birth. It is important to note that this article received a low risk of bias score through the Cochrane "RoB 2" tool, providing greater reliability to its results, which also align with the Michalowicz *et al*. 2006 (13) study mentioned earlier.

In studies where SRP was not performed on the experimental group but advanced oral hygiene instructions with cetylpyridinium chloride (CPC) (9,10) mouthwashes were given, preterm birth rates were also varied between control and experimental groups.

Jiang *et al*. in 2016 (10) found that the experimental group had a slightly higher preterm birth rate than the control group, possibly due to poorer oral health in the experimental group. These results correspond with a systematic review and meta-analysis by Polyzos *et al*. in 2010, which found an odds ratio of 1.15 (95% CI: 0.95 - 1.40), indicating a higher incidence of preterm birth in experimental groups (14).

In the study by Parry *et al*. in 2023 (9), the advanced oral hygiene protocol combined CPC mouthwash at 0.07% with fluoride toothpaste at 0.454%. Although the mean gestational age was lower in the experimental group, more cases of preterm birth were found in the control group, especially among unemployed patients, as this study aimed to relate preterm birth incidence in pregnant women diagnosed with periodontal disease to their socioeconomic status, which in turn is related to access to health education (9). A randomized clinical trial by Oo *et al*. in 2022 found no significant differences between the efficacy of CPC and CHX mouthwashes, however, CPC's lower cost may make it a good treatment option for low-income populations and it has fewer side effects (15).

The results obtained in this study are consistent with those found in other meta-analyses, such as the one conducted by Vergnes *et al*. in 2007, which concluded that although periodontal disease could be an independent risk factor for preterm birth, it is "overestimated" (16) due to higher-quality studies not supporting this association. Another systematic review by Boutin *et al*. in 2012 concluded that "there is not enough evidence to establish that SRP as a sole treatment during pregnancy is effective in reducing the incidence of preterm birth in women diagnosed with periodontal disease (17).

Beck *et al*. in 2019 and Xiong *et al*. in 2011 agree that a large number of randomized clinical trials conducted in recent years, despite having high methodological quality, have not related maternal periodontal treatment to reduced incidence of preterm birth, possibly due to the tendency to perform treatment during the second trimester of pregnancy (3,18), which is a very short period to reduce both local and systemic inflammation and its consequences (especially the presence of inflammatory mediators inducing labor) and achieve a true reduction in adverse pregnancy outcomes, with treatment ideally starting even a year before conception.

Daalderop *et al*. in 2018 indicate that there is now sufficient evidence to consider a relationship between maternal periodontal disease and various adverse pregnancy outcomes, especially preterm birth, and therefore, more epidemiological studies and systematic reviews in this area should be conducted (19).

## Conclusions

There is no demonstrated association between periodontal disease in pregnant women and the incidence of preterm birth. On the other hand, there is a slightly favorable trend towards non-surgical periodontal treatment during pregnancy as a measure to reduce the incidence of preterm birth, but it’s not statistically significant. To obtain a definitive answer, more randomized clinical trials following similar study and design parameters are needed.

## Figures and Tables

**Table 1:** Description of the selected studies.

Author	Experimental group	Control group	PD	PTB	Characteristics	Description
Caneiro *et al.*(6) 2019 Spain	n = 20	n = 20	periodontal pockets with a probing depth ≥ 3 mm in ≥ 2 teeth.	< 37 weeks GA.	• Caucasian race and between 18 to 40 years old. • Singleton pregnancies of ≥16 weeks gestation. • At least 20 natural teeth and diagnosed with PD. • No history of PTB. • No systemic diseases.	"To determine whether non-surgical periodontal treatment provided during pregnancy affects gestational age at delivery."
Jiang *et al.*(10) 2016 China and USA	n = 217	n = 212	periodontal pockets with a probing depth between 3.5 - 5.5 mm.	< 37 weeks GA.	• Older than 18 years of age. • Less than 20 weeks gestation. • No other severe oral conditions. • No systemic diseases or pharmacotherapy.	"To evaluate whether the use of 0.7% cetylpyridinium chloride mouthwash during pregnancy reduces the risk of APOs in a rural area of China."
Merchant *et al.* (8) 2018 USA	n = 413	n = 410	- mean clinical attachment loss.	< 37 weeks GA.	• Older than 16 years of age. • Singleton pregnancies between 6 days and 16 weeks gestation. • At least 20 natural teeth. • Diagnosed with PD. • No need for antibiotic prophylaxis before periodontal treatment.	"To evaluate how disparity in fetal survival between groups in the baseline study affected their outcomes due to bias, considering the Survival Average Causal Effect (SACE)."
Parry *et al.*(9) 2023 USA	n = 302	n = 311	moderate to severe gingivitis. Presence of ≥ 30 bleeding points on probing.	< 37 weeks GA.	• Singleton pregnancies between 8 and 24 weeks gestation. • At least 20 natural teeth. • ≥ 30 bleeding points. • No other severe oral conditions. • No systemic diseases or pharmacotherapy.	"To evaluate the efficacy of an advanced oral hygiene regimen on the occurrence of APOs in women with moderate to severe gingivitis in populations with different socioeconomic levels, particularly in groups with limited access to healthcare services."
Penova-Vaselinovic *et al.* (7). 2015 Australia	n = 40	n = 39	presence of periodontal pockets in at least 25% of sites with a probing depth ≥ 3.5 mm.	< 37 weeks GA.	• Older than 16 years of age. • Singleton pregnancies between 12 and 20 weeks gestation. • No fetal abnormalities. • No systemic diseases or pharmacotherapy. • No previous periodontal treatments. • At least 20 natural teeth.	"To investigate the effect of PD treatment in the second trimester of pregnancy on inflammatory mediators and clinical parameters in women with a clinical diagnosis of PD and to determine if these dental parameters or the inflammatory mediator profile in crevicular fluid can predict APOs such as PTB."

**Table 2:** Results obtained in the selected studies.

Author	Randomized clinical trial	Experimental group	Control group	Follow-up	ODDS RATIO	OR	CI	Results
Caneiro *et al.*(6). 2019 Spain	Yes	2 sessions of SRP before 24 weeks GA.	oral hygiene instructions.	16 weeks, 22 weeks, and 33 weeks GA.	0.28 95% CI (0.02 - 2.98)	OR < 1: lower probability of PTB in the experimental group.	CI includes 1: insufficient evidence.	"There were no statistically significant differences between the parameters of both groups. PTB rates were slightly lower in the experimental group, but not significantly."
- Jiang *et al.* (10). - 2016 China and USA	Yes	oral hygiene instructions and use of 0.7% CPC mouthwash for 30 seconds twice a day.	traditional oral hygiene instructions.	probing depth.	1.59 95% CI (0.51 - 4.92)	OR > 1: higher probability of PTB in the experimental group.	CI includes 1: insufficient evidence.	"There were no significant differences between the two groups regarding PTB, although it was slightly higher in the experimental group, whose population had worse periodontal health. The experimental group had a lower rate of PROM than the control group."
Merchant *et al.* (8). 2018 USA	Yes	oral hygiene instructions and 4 sessions of SRP before 21 weeks GA.	oral examination.	probing depth.	0.94 95% CI (0.48 - 1.82)	OR < 1: lower probability of PTB in the experimental group.	CI includes 1: insufficient evidence.	"When analyzing PTB and SAB values in each group, it was found that the experimental group had lower rates of PTB, intrauterine fetal demise (IUFD), and spontaneous abortion (SAB) despite having higher risk factors for PTB, such as hypertension, diabetes, and smoking."
Parry *et al.* (9). 2023 USA	Yes	advanced oral hygiene with 0.454% SnF2 toothpaste and 0.07% CPC mouthwash twice a day.	traditional oral hygiene twice a day.	Bleeding index and probing depth evaluated once a month for 3 months.	1.63 95% CI (0.903 - 3)	OR > 1: higher probability of PTB in the control group.	CI includes 1: insufficient evidence.	"There was a higher risk of PTB in the control group, with a higher mean gestational age in the experimental group, although there were no statistically significant differences between the groups. Unemployed patients in the control group showed the highest risk of PTB."
Penova-Vaselinovic *et al.* (7). 2015 Australia	Yes	3 sessions of SRP between 20 and 28 weeks of gestation.	same treatment post-delivery.	Gingival crevicular fluid (GCF) sample at 20 and 28 weeks of gestation.	0.80 95% CI (0.20 - 3.23)	OR < 1: lower probability of PTB in the experimental group.	CI includes 1: insufficient evidence.	"The levels of several inflammatory mediators found in GCF were lower in the experimental group than in the control group. However, no significant data were found on the benefits of the treatment in reducing the risk of PTB."
Parry *et al.* (9). 2023 USA	Yes	advanced oral hygiene with 0.454% SnF2 toothpaste and 0.07% CPC mouthwash twice a day.	traditional oral hygiene twice a day.	Bleeding index and probing depth evaluated once a month for 3 months.	1.63 95% CI (0.903 - 3)	OR > 1: higher probability of PTB in the control group.	CI includes 1: insufficient evidence.	"There was a higher risk of PTB in the control group, with a higher mean gestational age in the experimental group, although there were no statistically significant differences between the groups. Unemployed patients in the control group showed the highest risk of PTB."
